# Chronic Electromagnetic Exposure at Occupational Safety Level Does Not Affect the Metabolic Profile nor Cornea Healing after LASIK Surgery

**DOI:** 10.1155/2014/762364

**Published:** 2014-03-18

**Authors:** David Crouzier, Vincent Dabouis, Edgar Gentilhomme, Rodolphe Vignal, Fréderic Bourbon, Florence Fauvelle, Jean-Claude Debouzy

**Affiliations:** ^1^IRBA, Unité des Rayonnements Non Ionisants et de Biophysique, BP 73, 91220 Brétigny sur Orge, France; ^2^IRBA, Service d'Imagerie et de Microscopie, BP 73, 91220 Brétigny sur Orge, France; ^3^Hopital des Armées Bouffard, Service d'Ophtalmologie, 85024 Djibouti, Djibouti

## Abstract

LASIK eye surgery has become a very common practice for myopic people, especially those in the military. Sometimes undertaken by people who need to keep a specific medical aptitude, this surgery could be performed in secret from the hierarchy and from the institute medical staff. However, even though the eyes have been previously described as one of the most sensitive organs to electromagnetic fields in the human body, no data exist on the potential deleterious effects of electromagnetic fields on the healing eye. The consequences of chronic long-lasting radar exposures at power density, in accordance with the occupational safety standards (9.71 GHz, 50 W/m^2^), were investigated on cornea healing. The metabolic and clinical statuses after experimental LASIK keratotomy were assessed on the different eye segments in a New Zealand rabbit model. The analysis methods were performed after 5 months of exposure (1 hour/day, 3 times/week). Neither clinical or histological examinations, nor experimental data, such as light scattering, ^1^H-NMR HRMAS metabolomics, ^13^C-NMR spectra of lipidic extracts, and antioxidant status, evidenced significant modifications. It was concluded that withdrawing the medical aptitude of people working in electromagnetic field environments (i.e., radar operators in the navy) after eye surgery was not justified.

## 1. Introduction

LASIK surgery is routinely used to correct refraction abnormalities, such as myopia, hypermetropia, or astigmatism, without wearing glasses or contact lenses [1]. This surgery consists of creating a 100–120 micron-thin flap and then sculpting the cornea with an excimer laser to build a new curvature that would correct the optical defect. This act is painless and only needs a short period for recovery [2]. In the military, this surgery is presently not admitted for some specific job positions. However, this intervention is sometimes secretly realized for people who need to maintain their medical aptitude. This is particularly true for the navy and the air force workers whose job absolutely needs good visual acuity, for example, in optical survey tasks. However, although most of these people do not present any medical contraindication to return to their position after surgery, others are exposed to concomitant aggressive environments, such as professional exposure to electromagnetic fields.

The eyes are known to be one of the most sensitive organs in the human body to electromagnetic field exposure. This specific susceptibility has been widely described and is mainly related to the low level of eye vascularization leading to low heat dispersion. This link was identified quickly, especially by considering links between cataracts and professional microwave exposure (radar operators) [3–5]. Many studies have shown that high power electromagnetic field exposure could lead to a significant temperature increase in the eye that is able to induce cataracts [6–8]. These effects have been used for the establishment of international protecting guidelines (International Commission on Non-Ionizing Radiation Protection (ICNIR) [9]) and others in north America (Institute of Electrical and Electronics Engineers (IEEE) [10]).

Besides these well-known effects, more recent works have dealt with the potential hazardous nonthermal effects of radiofrequency on the eyes. Although no mechanism has been clearly exposed, some authors have identified deleterious effects in different parts of the eye, such as the lens [11–13], the cornea [14, 15], or the retina [15]. Such results were frequently related to an oxidative stress increase [16]. However, due to the lack of reproducibility, those results are still under discussion.

The healing eye, after situations like ocular wounds or repair following surgery, appears to be an even more sensitive organ. Scars on the cornea and refractive modification could occur, as for light and electromagnetic field dispersion, and could lead to a local specific absorption rate (SAR) increase. Lens opacities and transparent intraocular media composition would also result in local energy absorption modifications.

To our knowledge, no previous works have focused on the deleterious effects of electromagnetic fields on a healing eye after wounding or refractive surgery. By considering the other eye-aggressive agents such as UV and diabetes, corticotherapy can lead to an increase in the promotion of reactive oxygen species and to metabolic changes in the different eye targets [17], such as the lens, and vitreous [18] and aqueous humor; these works focused on metabolic changes after chronic radiofrequency (RF) exposure. In this paper, LASIK surgery was performed on New Zealand rabbits to obtain a realistic model of the healing eye. As ametropy correction was not of interest in this work, only keratotomy was performed without any stromal photoablation. One month after surgery, animals were exposed for one hour, 3 times a week for 5 months to a 9.71 GHz electromagnetic field at a power density of 50 W/m^2^, mimicking chronic occupational exposure to pulsed RF emitted by a radar. The day following the last exposure, standard ophthalmologic exams were performed. After euthanasia, specific attention was drawn to the lens. The transparency was assessed by light scattering, and histological analysis was performed on lenses and corneas. The lens metabolism was evaluated by using High Resolution Magic Angle Spinning (HR-MAS) NMR. HR-MAS NMR is now considered one of the reference methods that can give a metabolomic profile without any sample preparation of the biopsied tissues [19–21] and has successfully been used in lens metabolism analysis [22]. In addition, the lipid composition was studied by liquid high resolution NMR on standard lipid extracts. Finally, the oxidative stress, as potential mechanistic support to ocular effects of electromagnetic exposure, was measured in plasma and vitreous and aqueous humor.

## 2. Materials and Methods

### 2.1. Animal Experiments

All procedures were in accordance with the standards for animal care established by the Army Biomedical Research Institute (IRBA) and were approved by the IRBA ethics committee for animal experimentations (decree 87-848 19 October 1987, edited by the French government).

60 male New Zealand rabbits (Elevage Charles River, France), weighing 2.5–3 kg upon arrival, were used. Animals were housed 1 per cage at control room temperature (21°C) with 12 h/12 h light-dark cycle (dark period from 6 p.m. to 6 a.m.). Food and water were available ad libitum. The animals were randomly assigned to four different groups: surgery/exposed, surgery/sham exposed, sham surgery/exposed, and sham/sham.

### 2.2. Surgery

Anesthesia was obtained by intramuscular injection of Zolazepam/Tiletamine 10 mg/kg (Zoletil 100, Merieux, France). In addition, topical bupivacaine hydrochloride 1% (Roche, France) was applied to each eye just before surgery. Bilateral keratotomy was realized using a one-use Moria microkeratome, set to cut a flap 8.5 mm in diameter and 100 *μ*m in thickness. The system was fitted to the rabbit cornea by removing the mechanical forward moving system. The microkeratome base was pressed firmly onto the cornea, the suction system was activated, and the microkeratome head was manually handled to create the flap. The flap reflected its hinge against the conjunctiva. The flap and stromal bed were irrigated with balanced salt solution (BBS, Alcon). The flap was replaced in its original position without any photo laser ablation. After surgery, animals had a 1 week postoperative recovery period with an everyday topical instillation of antibiotic (Rifamycin) and antiseptic-steroidal anti-inflammatory Tobramycin/Dexamethasone eye lotion (Tobradex, Alcon). In order to limit flap removal and eye scratching, each animal wore an Elizabethan collar during this period. Rabbits with flaps that were displaced or with visible striae after surgery were excluded from the study.

### 2.3. Exposure Condition and Dosimetry

Exposure devices were based on a pulsed X Band ESR spectrometer, ESP 380 system (Brüker, France) with a 1 kW travelling wave-tube amplifier (TWT). The TWT output was directly connected to a horn antenna (length 17 cm, gain 20 dB) located in an anechoic chamber. The system emitted a typical pulsed electromagnetic field at 9.71 GHz, with a pulse length of 9000 ns and a 1% duty cycle.

During exposure, the animal was put in polycarbonate restrainers, located in the anechoic chamber at 30 cm from the end of the antenna. According to their group, animals were then exposed or sham exposed 1 month after surgery for 1 h/day, 3 days a week for 5 months, at a power density of 50 W/m², which is the ICNIRP exposure recommendation for workers.

The electromagnetic exposure conditions were controlled and measured before each experiment using a multifrequency anisotropic field meter (FP7050, AR worldwide, USA).

The specific absorption rate (SAR) was estimated by direct measurement of temperature increase at higher power density. It was derived according to the following equation: SAR = *C*(*dT*/*dt*), where *C* is the thermal capacity in J/K/kg, *dT* is the temperature variation in *K*, and *dt* is the time variation in seconds. In such conditions, maximal SAR value in the eye was 6.35 W/Kg and no temperature increase could be measured.

### 2.4. Clinical Observation/Euthanasia

Clinical ophthalmologic examination was performed the day after the surgery, one week after, one month after (before the first RF exposure), and then at three and six months (before euthanasia). This exam was performed with an ophthalmoscope and consisted of the observation of any anomalies of the cornea, the lens, and the fundus oculi. The integrity of the cornea was assessed by fluorescein examination.

At the end of the experiment, before euthanasia, blood samples were collected in lithium heparin sample tubes for oxidative stress analysis. The animals were euthanized by a lethal injection of pentobarbital (Dolethal, Vetoquinol, France). Aqueous humor was punctured and immediately frozen in liquid nitrogen for oxidative stress analysis. The corneoscleral rims were removed with Westcott scissors. One cornea was used for histological analysis and then placed in a 4% formaldehyde solution; the other was used for NMR analysis and immediately frozen in liquid nitrogen. After light scattering measurement, lenses were frozen for NMR analysis.

### 2.5. Light Scattering

**Figure 1:**
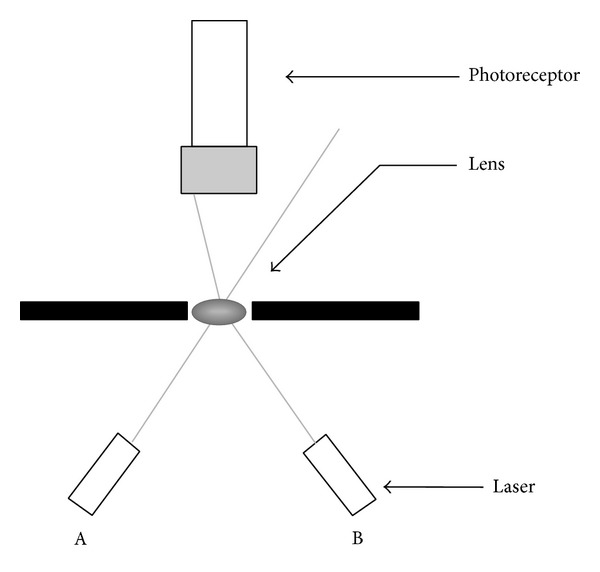
Light scattering principle. Position A represents the light path through a normal lens, while position B represents the light path through a lens with diffraction abnormalities.

The optical quality of the lens was assessed by light scattering, as described by Söderberg et al. [23]. Briefly, the device developed for the measurement of light dissemination was composed of an illumination source and a photometry unit (Figure 1).

After euthanasia, the lens was removed from the eye and immediately placed in a dark measuring chamber. The beam of the 670 nm diode laser located at 30 mm goes through the lens with a 45° incidence angle. If the lens is damaged, its ability to focus the laser beam at the various locations is altered, leading to an increase in the light diffusion.

### 2.6. Oxidative Stress

The quantification of oxidative stress was assessed in blood serum and in aqueous humor by using an Antioxidant Assay Kit (Caymann Chemical, USA), according to the manufacturer's instructions. This kit measures the total antioxidant capacity. The assay relies on the ability of the antioxidant in the sample to inhibit the oxidation of ABTS to ABTS+ by myoglobin. Trolox, a water-soluble tocopherol, is used as a standard; the antioxidant capacity of the sample is quantified as millimoles of Trolox equivalents. The optical density of each well was measured using a microtiter plate fluorometer Mithras LB 940 at 405 nm (Berthold Technologies, Bad Wildbad, Germany) and processed using the MikroWin software.

### 2.7. Liquid NMR

All high-resolution NMR spectra in liquid solution were recorded on a Bruker AM400 spectrometer (9.4T) operating at 400.13 MHz for protons.

^1^H-NMR spectra from aqueous and vitreous humors were recorded after lyophilization of the samples. The pellets were suspended in 500 *μ*L D_2_O and placed in a 5 mm NMR tube. The temperature of the samples was kept at 25°C using a Bruker BVT-2000 temperature regulation unit. A simple presaturation of the water proton resonance was applied before acquisition (1 sec). 32 K data points were collected on a spectral width of 10 ppm. 256 scans were used for each spectrum. A line broadening of 0.2 Hz was also applied before Fourier Transform. The attributions of the resonances were drawn from the literature and from classical NMR methods (COSY, TOCSY, HMQC [24]).

^13^C-NMR experiments on lenses and corneas were recorded on the lipidic phase of methanol/chloroform/water (2/1/0.8 V/V/V) extracts obtained according to the method of Bligh and Dyer [25]. The lipid phase was evaporated under nitrogen flux and resuspended in 500 *μ*L of deuterated chloroform/methanol 4/1 V/V. Chemical shift reference was the central peak of chloroform, set at 77 ppm. Peak assignment was realized following Husted et al. [26] and classical NMR methods [24]. The spectra were recorded at 100.6 MHz, using a composite proton decoupling, a spectral width of 250 ppm, a recycling delay of 2 sec, and 64 K data acquisition points. 40,000 scans per sample were required to enhance the signal-to-noise ratio. When possible, the single resonances were integrated and normalized by the whole integral spectrum (excluding solvent resonances). For cornea experiments, signal-to-noise ratio was not sufficient to allow direct peak intensity measurements, and it was necessary to perform a bucketing of the spectra, that is, to cut the spectrum into sections, the magnitude of which was then used as index after normalization by the total spectrum magnitude.

For the spectra of lenses, the signal-to-noise was of better quality and allowed to measure and normalize peak intensities for the different resonances used to build the index to compare groups. It should be noted that the normalization of the data used the sum of the integrals of the 180–100 ppm, 75.5–49.7 ppm, and 45–10 ppm regions to avoid dramatic errors related to the intense solvent (methanol and chloroform) resonances.

The selected peaks of interest are shown in Table 1; these data were then used to build the index shown in Table 2.

### 2.8. HRMAS

Immediately after the light scattering measurement, the rabbit lenses were frozen in liquid nitrogen. The samples were then stored at −80°C to ensure conservation before NMR experiments.

For HRMAS NMR analyses, a 15 mg fragment was obtained by cutting the lenses with a surgical blade. The shape of the biopsy was a circular coin, which was obtained by using a solid matrix guiding the blade. Such samples contained almost all of the different parts of the lenses, from the inside to the external layer. Such pieces were transferred into a HRM NMR 50 *μ*L rotor (in ZrO2). Rotor filling was done with D_2_O solution of TSP (tetra silyl propionate) 1 mM to ensure both field lock and chemical shift reference (the chemical shift of TSP was set to 0 ppm). After sealing, the rotor was introduced into the NMR magnet, the temperature of which was maintained at 4°C all along the experimental time.

^1^H-HRMAS NMR data were recorded at 400.13 MHZ on a Bruker Avance (Bruker Biospin, Wissembourg, France) NMR spectrometer. A modified spin-echo sequence (Carr-Purcell Meiboom Gill) was used: the echo had to be synchronized with the sample rotation speed (4 kHz). The 30 ms echo time allowed minimization of the contributions to the spectra of broad macromolecules and lipidic resonances. Π-pulses were separated by 250 *μ*s. A 2-second presaturation of the intense water contribution was also required. By using a recycling delay of 3.5 second and 256 scans per spectrum, the total acquisition time for a given sample was 15 min.

### 2.9. Histology

Corneas from control or treated groups were fixed with 3.7% formaldehyde (Sigma Aldrich, Saint Louis, Missouri, USA) in a sodium phosphate buffer solution at a pH of 7.4. After paraffin embedding, 5 *μ*m sections were cut and stained with hemalum phloxine saffron (HPS) for histological evaluation.

### 2.10. Statistical Analysis

Classical tests: all results are presented as mean ± SEM. The 4 different exposure conditions were randomized: surgery/exposed, surgery/sham exposed, sham surgery/exposed, and sham/sham. Statistical comparisons were achieved using nonparametric tests.

Statistics for PCA, PLS-DA: here, the populations were gathered in “exposed” versus “nonexposed (sham)” classes following the results of nonparametric tests. The NMR data then had to be processed with the Bruker Xwin-NMR v2 software. The spectra were apodized by using a 0.3 Hz Lorentzian convolution prior to Fourier transformation. Baseline correction was also automatically realized. The final spectra were finally segmented in spectral bands of 0.2 ppm (AMIX v3.5 software, Bruker-Biospin, France). These bands were then statistically analyzed with SIMCA v12 (Umetrics, Umea, Sweden); a principal components analysis (PCA) was first performed to identify possible gathering data and unclassified aberrant criterions. PLS-DA (Projection to Latent Structure-Discriminant Analysis) was finally built to maximize intergroup variance and to minimize the intragroup variance. This processing assumed that a given result to the sham or exposed groups was realistic.

## 3. Results

### 3.1. Global Observations: Clinical Ophthalmologic Examination and Light Diffusion

Observation of the periocular area and of the cornea did not show any inflammation, edema burns, or conjunctivitis in any case. However, if no keratitis, corneal opacity, or traces of surgery were found, limited hazes were detected in two cases on exposed rabbits. Ophthalmoscope examination showed neither lens opacity nor eye floaters within the vitreous humor, meaning that there was no major cataract formation or vitreous degeneration.

**Figure 2:**
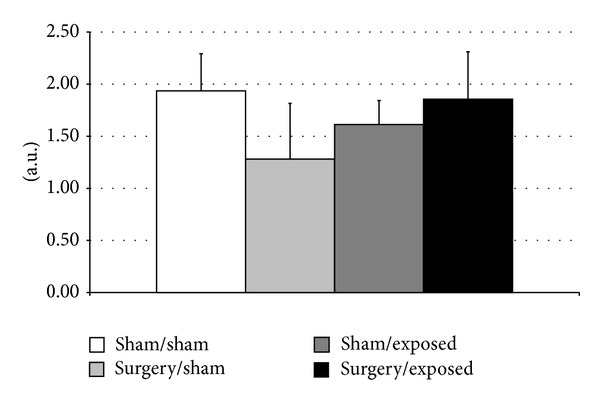
Light scattering diffusion. For each group, the value was the mean of 15 measures ± SD. Significance was determined using the Mann-Whitney* U* test.

This examination was completed by light diffusion measurements of the lens, as shown in Figure 2. The standard deviation of each group shows an important dispersion of light scattering properties in the same group. However, no significant difference was found between the four groups or between the exposed and sham-exposed groups after gathering the surgery and sham surgery groups, by using nonparametric tests (Mann Whitney).

### 3.2. Cornea: Histology and ^13^C-NMR on Lipidic Extract Results

#### 3.2.1. Histology

**Figure 3:**
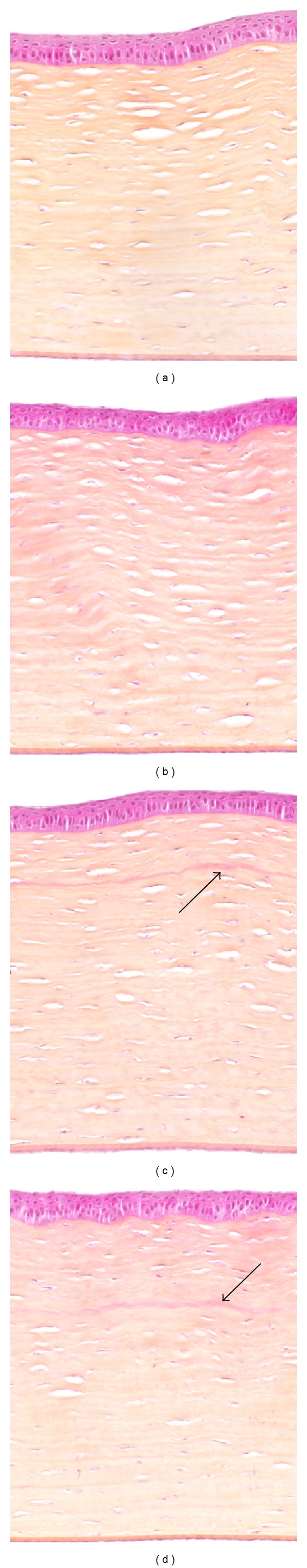
Histological aspect of the cornea in the sham/sham exposed group (a), sham surgery/exposed group (b), surgery/sham exposed group (c), and surgery/exposed group (d).

Six months after surgery, the aspect of nonexposed operated corneas (Figure 3(a)) has been restored, similar to control corneas (Figure 3(b)). The epithelium was regular, with four or five nonkeratinized cellular layers adherent to the Bowman's membrane. The stroma was homogenous, with parallel collagen plates and cells disposed upon the Descemet's membrane and the endothelium. One could only notice a fine colorful linear mark corresponding to the surgical scare and a slight thickening of the epithelium at the edge of the flap.

After six months of exposure, slight modifications of the epithelium were noticed. The epithelial aspect was less regular with limited proliferative areas (Figure 3(c)). The stroma and endothelium were not modified.

When exposures were performed after surgery (Figure 3(d)), the proliferative areas were found to be more numerous, while the other tissues of the cornea, stroma, and endothelium appeared unmodified, as in the control groups.

#### 3.2.2. ^13^C-NMR Analysis of Cornea Lipid Extracts

**Figure 4:**
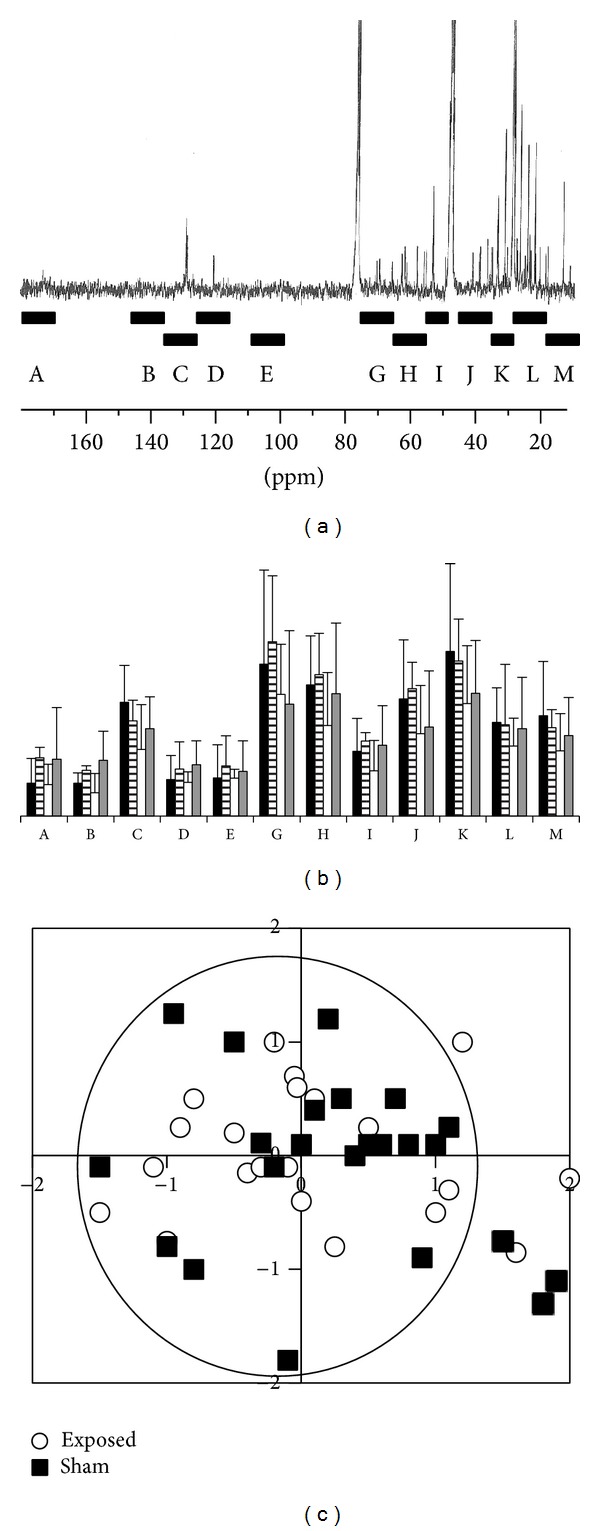
^13^C-NMR spectrum of cornea lipid extract in CDCl_3_/MeOD. Buckets are represented by black boxes and identified, as used in statistical analyses (a). Histogram representation of Kruskal-Wallis tests, using four classes: surgery/exposed (black box), surgery sham/exposed (strip box), surgery/sham exposed (light grey box), and sham surgery/sham exposed (white box) (b). PLS-DA correlation circle for type two tests: black boxes represent the sham-exposed groups; white circles represent the exposed groups (c).

The spectrum in Figure 4 is typical of the lipid extract of biological tissues, where different resonances can usually be easily attributed to different chemical groups [27]. However, due to the poor signal-to-noise ratio and experimental time constraints, a statistical analysis could only be performed after having bucketed the spectra, as seen in the ^13^C-NMR trace of Figure 4(a). Note that buckets usually covered a 10 ppm spectral range except: for the I area (50–55 ppm) to avoid the methanol (solvent) contribution at 48 ppm and for the K area (30–35 ppm) to clearly separate the intense methylenic contribution (20–30 ppm). Besides, some areas containing no meaningful resonance were discarded from analysis (i.e., the 77–100 ppm). After area integration and normalization (see methods), the 4 different classes were compared in pairs, as presented in Figure 4(b). The absence of any significant difference between groups and also great interindividual variability were drawn from the nonparametric analysis (Kruskal Wallis) and confirmed by the PLS-DA analysis, as observed in the correlation circle presented in Figure 4(c) (in the case of type 2 tests). The histological analysis of cornea slices also failed to show any morphological difference at the tissue level.

#### 3.2.3. Aqueous Humor: ^1^H-NMR Metabolic Analysis and Oxidative Status

**Figure 5:**
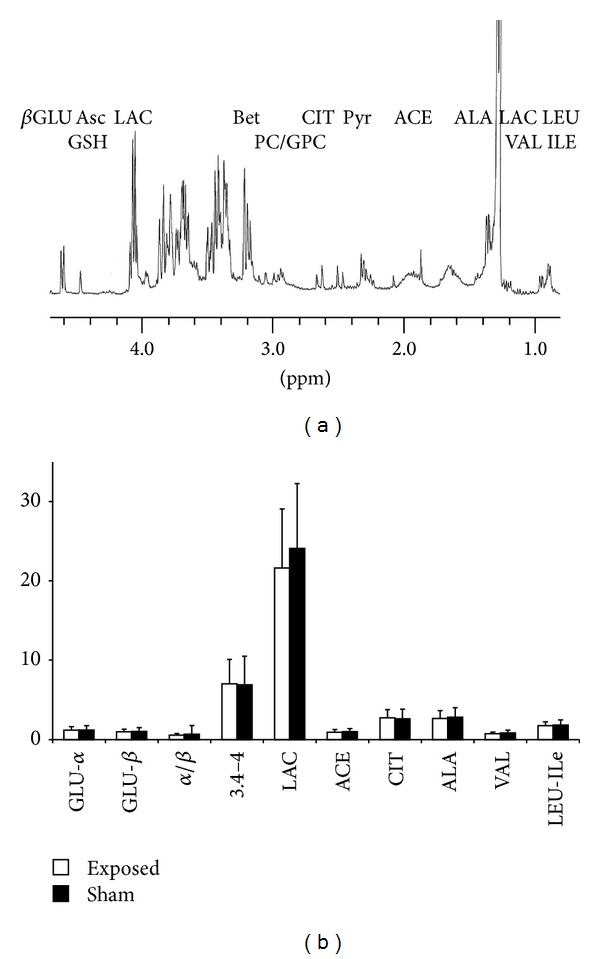
^1^H-NMR spectra of aqueous humor (a). A two-class analysis of metabolites contributions to ^1^H-NMR spectra of aqueous humor, sham-exposed groups (white) versus exposed group as is shown on the bottom trace (b). Meanings of abbreviations: GLU-*α*: glucose *α*, GLU-*β*: glucose *β*, LAC: lactate, ACE: acetate, CIT: citrate, ALA: alanine, VAL: valine, and LEU-ILE: leucine-isoleucine.

^*1*^ *H-NMR Metabolic Analysis*. Despite several broad lipidic and macromolecular contributions (especially around 1.6 ppm and 2 ppm), ^1^H-NMR spectra recorded on aqueous humor (Figure 5(a)) allowed the identification and integration of numerous resonances of soluble metabolites. However, due to the severe overlaps occurring in the 3.4–4 ppm area (mainly related to intense glycidic and polar head groups of phospholipid contributions), this spectral region was considered as a whole and integrated as so. After a four-class analysis (similar to that used in the previous section) using paired KW tests, no significant difference was identified between the exposed versus sham classes, as shown in Figure 5(b). Also here, no difference was evidenced in amino acid contributions (Leu, Ile, Val, Ala), metabolic intermediates (LAC, Cit, ACE), glucose contributions (*α*- and *β*-glucose contributions), or relative proportions (as measured on H1 anomeric resonances).

**Figure 6:**
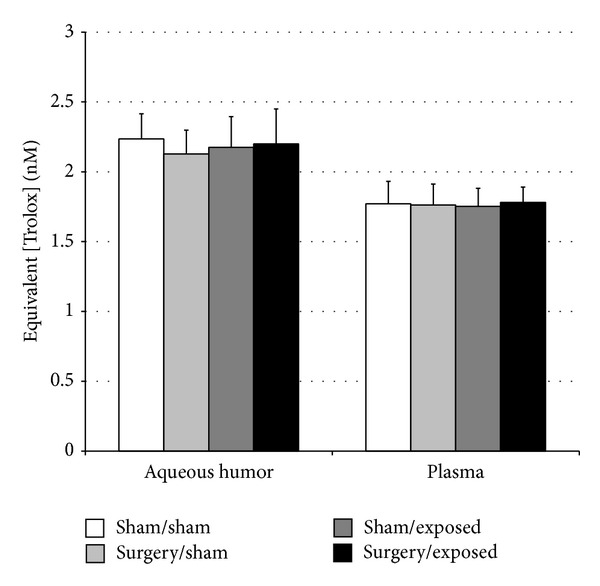
Oxidative stress for the 4 different groups measured in aqueous humor and plasma. For each group, the value was the mean of 15 measures ± SD. Significance was determined using the Mann-Whitney* U* test.

*Oxidative Stress*. Oxidative stress was assessed in the aqueous humor and in the plasma by measuring reserve in the overall remaining antioxidant capacity. The results are shown in Figure 6 and are expressed as the equivalent concentration of Trolox used to have the same antioxidant effect. Six months after surgery and following 5 months of chronic electromagnetic exposure, no significant difference was observed between groups either in the aqueous humor or in plasma.

#### 3.2.4. Lenses

**Figure 7:**
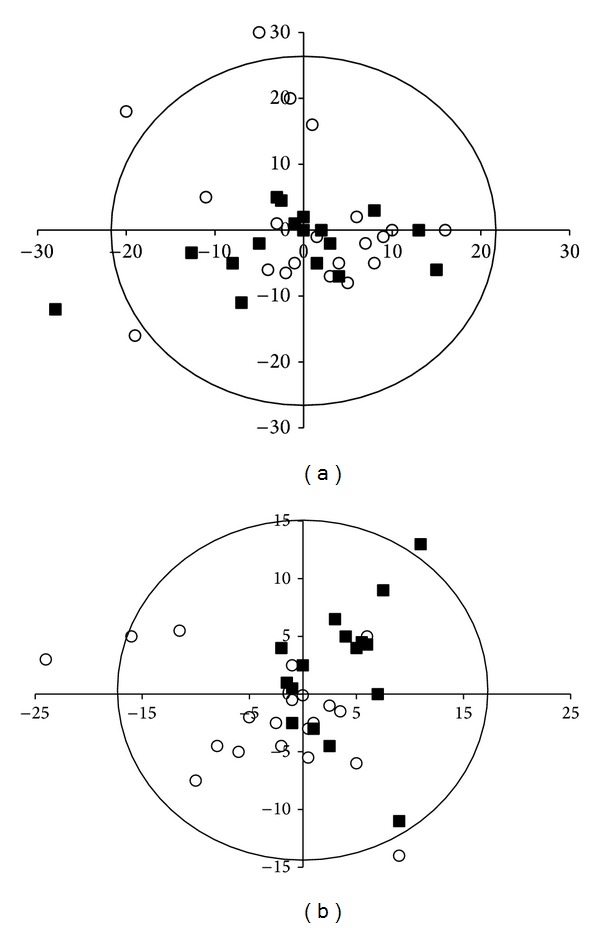
Lenses. PLS-DA correlation circle for type 2 tests: shams, black squares versus exposed, white squares. (a) The PCA correlation circle was built using 8 main components. (b) PLS-DA construction was built from two main components.

*NMR-HRMAS: Metabolic Fingerprint*. The PCA correlation circle shown in Figure 7(a) was built using 8 main components. This explained only 40% of the total system. A PLS-DA construction (circle shown on Figure 7(b)) was then built from the two main components, yielding a quality factor of* R2Y* = 0.3. Besides the factor of predictability, a* Q2* of −0.2 was found. As a given model is considered robust for* R2Y* > 0.5 and* Q2* > 0.5, it was concluded that sham and exposed populations could not be distinguished by this method from ^1^H-HRMAS analysis of lenses.

**Figure 8:**
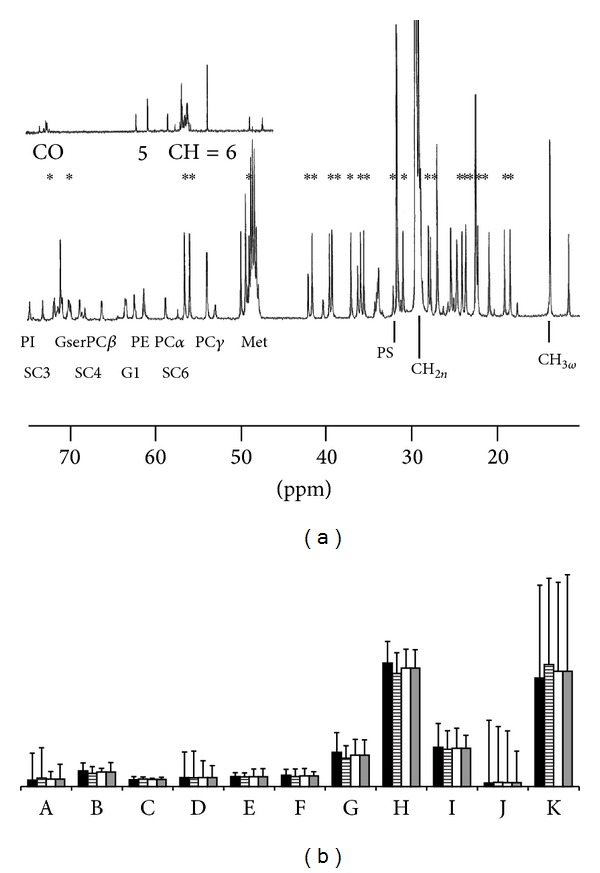
(a) ^13^C-NMR spectrum of lenses lipid extract in CDCl_3_/MeOD. Cholesterol resonances are labeled with stars, and other resonances are labeled as mentioned in the methods section. (b) Comparative histogram representation of Kruskal-Wallis tests, using four classes: surgery/exposed (black box), surgery sham/exposed (strip box), surgery/sham exposed (light grey box), and sham surgery/sham exposed (white box). A, chain length; B, chain unsaturation; C, chain to glycerol ratios; D, relative contribution of PS to phospholipids; E, relative contribution of PI to phospholipids; F,  relative contribution of PC to phospholipids; G, relative contribution of PE to phospholipids; H, relative contribution of PEPL to phospholipids; I,  relative contribution of glycidic moieties to total polar head groups; J,  cholesterol/phospholipid ratio; K, cholesterol/phospholipids.

*Lipid Profiles of Lenses Extracts:* ^*13*^ *C-NMR*. The same experimental procedure was used as for cornea analysis. Here, however, the signal-to-noise ratio was sufficient to allow the identification of numerous peaks. Many of them (identified by stars in Figure 8(a)) were related to cholesterol resonances. For cholesterol contribution analysis, C5, C6, and C17 resonances were used (located at 142, 122, and 56.5 ppm, resp.). Besides, the contributions of the different resonances of lipidic chains (methyl *ω* or *ω*-3, methylenic, methynic, carboxylic…) or head groups of phospho- or glucolipids (mainly in the 50–75 ppm part of the spectrum) could be identified and measured to build the different indexes shown in Figure 8(b) and described in the figure captions.

Here, also, no significant difference was found between groups taken separately by pairs or gathered in exposed versus sham groups.

**Figure 9:**
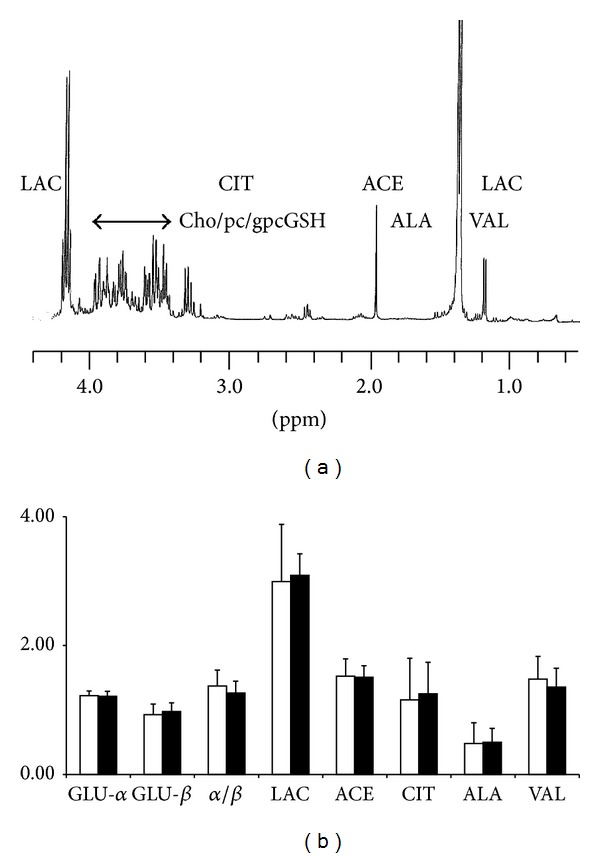
(a) ^1^H-NMR spectra of vitreous humor were lyophilized and resuspended in D_2_O. The glycidic area is labeled with an arrow; other metabolites are shown with their abbreviations. (b) Comparative histogram of metabolite contributions was extracted from ^1^H-NMR spectra of vitreous humor, sham-exposed groups (white) versus exposed groups. Meanings of the abbreviations: GLU-*α*: glucose *α*, GLU-*β*: glucose *β*, LAC: lactate, ACE: acetate, CIT: citrate, ALA: alanine, VAL: valine, and LEU-ILE: leucine-isoleucine.

*Vitreous Humor*.  Figure 9(a) shows the high-field part of the ^1^H-NMR spectrum of aqueous humor obtained after lyophilization and resuspension in D_2_O. Such a spectrum looks very similar to those recorded for aqueous humors, whereas the magnitudes of the different metabolites were found to be in very different proportions. The same analysis performed on these spectra evidenced no significant differences (see for instance the histograms in Figure 9(b)), while PLS-DA allowed no clear distinction between the different classes.

## 4. Discussion

The aim of this work was to investigate whether chronic electromagnetic field exposure of the eyes after keratotomy would influence healing or at least induce metabolic or structural modifications in the eyes. Such an interrogation was supported by several professional situations, such as those for radar technicians and engineers in airports or navy personnel aboard warships. The question here was to determine whether radar exposure under chronic conditions at occupational power levels could lead to deleterious effects regarding cornea healing or metabolic perturbations in the different building blocks of the eyes. That is why the experimental model chosen in this paper used New Zealand rabbits and eye exposures at 50 W/m^2^, 1 hr/day, 3 days/Week over 5 months, under pulse conditions mimicking radar exposure (duty cycle of 1/1000). The different experiments performed on the different parts of the eyes, cornea observations, analyses, and histology failed to find any significant effect. This was in strong agreement with papers underlining that significantly more intense power is requested to obtain cornea lesions [28, 29]. The question was clearly less intuitive about the liquid media of the eyes, in the aqueous and vitreous humors, since only recent metabolic studies have been performed on these media, mainly using NMR methods, generally under well-identified deleterious situations such as diabetes [30], or UV exposure [17]. In such cases, soluble metabolite and lipidic analyses similarly showed no difference under radiofrequency exposure.

The question was more subject to controversy in the case of lenses. On the one hand, there is no doubt today that microwave radiation via thermal effects may lead to cataracts. Hence, the international guidelines for exposure limits to microwave radiations were directly related to protection against thermal effects [9]. Former results with rabbits have shown [31] that the threshold intensity radiation for cataract formation under microwave exposure was about 100 minutes at 2.45 GHz, with a power density of 1.5 kW/m^2^ (i.e., 130 kW/kg peak absorption). Conversely, more recent works [11] have reported cataract induction when using longer exposure durations (8 days) at lower power levels (0.1 W/m^2^ at 1.1 GHz, to be compared to ICNIRP levels of 50 W/m^2^). In the work by Dovrat et al., the formation of bubbles inside the lens, close to the lens sutures, was identified. These results appeared very different from a local temperature increase, where bubbles are also created but appear more homogeneous and far from sutures. If no clear mechanism was identified, the author suggested that such effects could result from microscopic friction at the interface between two bundles of lens fibers causing a local temperature increase. One would notice that the SAR estimation was only extracted from the ratio of source power to eye weight (1.4 W/kg). Our study showed no difference between the sham groups and the exposed population (and also no difference with time within one of these groups). As the rabbits were still moving in the field, spatial and time averaging occurred; thus, an evaluation of SAR was not useful, and the power density was only considered around 90 W/m^2^ RMS at 10 GHz, the ICNIRP limit for professional exposure.

Due to some limitations, extrapolation of that work to the human eye should be carefully considered. The main limitation could be addressed on the surgical operation itself. The experimental constraints do not allow the realization of photoablation in our model. However, ablation of the stromal bed could add great stress caused by the laser energy absorption. Moreover, the rabbit eye presents some differences to the human eye, such as corneal endothelium with replicative capacity, unlike in the human cornea. All of those considerations lead to the potential underestimation of possible effects of human electromagnetic exposure.

## 5. Conclusion

This paper dealt with the consequences of chronic electromagnetic exposures at occupational levels on the eyes after keratotomy. From the extensive study of all compartments of the eye using various approaches, including anatomy, histology, and NMR lipidic and metabolic analysis, as well as light diffusion, no dramatic deleterious effect was identified on eye healing or eye structure under these conditions. However, with regard to the suggestion that people occupationally exposed to X-band radars (10 GHz) bear no risk in the workplace when their vision requires corrective keratotomy, further investigations are required to identify or inform the bubbling process observed here. This, for instance, would be based on variations of the power of exposure and duty cycles at very low levels or conversely at levels close to thermal effects. This work, involving numerous animals and long experimental periods, is currently in progress.

## Figures and Tables

**Table 1:** NMR peaks of interest.

Molecule	Proton nomenclature and chemical shift (ppm)	Label
Acyl chains		
Terminal methyl	tCH_3_ area 14.4–14.8	G
Chain methylenic	CH_2n_ area 29.8–31.2	H
Methynic	CH = area 128–131	I
Carboxylic	CO area 174–177	J

Cholesterol	Peak intensities	
C5	142.6	K
C6	121.7	L
C17	56.5	M

Sugar moieties of glucolipids	Peak intensities	
C1	104.1	N
C6	62	O

Phospholipid head groups	Peak intensities	
Sphingomyelin and phosphatidylcholine, PCSM	CH_3*γ*_ 54; CH_2*α*_ 60.3; CH_2*β*_ 67	P, Q, R
Phosphatidylserine, PS	CH_2*α*_ 56; CH_*γ*_ 172.3	T, U
Phosphatidylethanolamine, PE	CH_2*β*_ 41.4	V
Phosphatidylinositol, PI	CH_2_ 75	W
Phospholipid glycerol, PG	C1–C3 64-65 area	X
Plasmalogen, PEPL	C2′ 108; C1′ 145	Y, Z

**Table 2:** NMR index comparison. The letter in formula column refers to the label of the peak of interest.

Index	Formula
Chain length	(G + H + I + J)/G
Chain insaturation	I/(G + H + I + J)
Cholesterol/glycerol	(K + L + M)/X
Cholesterol/chains	(K + L + M)/(G + H + I + J)

Contribution to the phospholipids of	
Phosphatidylserine, PS	(T + U)/(P + Q + R + T + U + V + W + Y + Z)
Phosphatidylethanolamine, PE	V/(P + Q + R + T + U + V + W + Y + Z)
Sphingomyelin and phosphatidylcholine, PCSM	S/(P + Q + R + T + U + V + W + Y + Z)
Plasmalogen, PEPL	(Y + Z)/(P + Q + R + T + U + V + W + Y + Z)

Sugar to phospholipids ratio	(N + O)/(P + Q + R + T + U + V + W + Y + Z)
